# Can the Cytokine Profile According to ABO Blood Groups Be Related to Worse Outcome in COVID-19 Patients? Yes, They Can

**DOI:** 10.3389/fimmu.2021.726283

**Published:** 2021-10-13

**Authors:** Álvaro Tamayo-Velasco, María Jesús Peñarrubia Ponce, Francisco Javier Álvarez, Hugo Gonzalo-Benito, Ignacio de la Fuente, Sonia Pérez-González, Lucía Rico, María Teresa Jiménez García, Alba Sánchez Rodríguez, Milagros Hijas Villaizan, Marta Martín-Fernández, Carlos Dueñas, Esther Gómez-Sánchez, María Heredia-Rodríguez, Óscar Gorgojo-Galindo, Itziar Fernández, Lourdes del Río, Irene Carnicero-Frutos, María Fe Muñoz-Moreno, Eduardo Tamayo, David Bernardo, Pedro Martínez-Paz

**Affiliations:** ^1^ Hematology and Hemotherapy Service, University Clinical Hospital of Valladolid, Valladolid, Spain; ^2^ Department of Pharmacology, Faculty of Medicine, University of Valladolid, Valladolid, Spain; ^3^ BioCritic Group for Biomedical Research in Critical Care Medicine, Valladolid, Spain; ^4^ Research Unit of University Clinical Hospital of Valladolid, Institute of Health Sciences of Castile and Leon (IECSCYL), Soria, Spain; ^5^ Department of Internal Medicine, Faculty of Medicine, University of Valladolid, Valladolid, Spain; ^6^ Internal Medicine Service, University Clinical Hospital of Valladolid, Valladolid, Spain; ^7^ Department of Surgery, Faculty of Medicine, University of Valladolid, Valladolid, Spain; ^8^ Anesthesiology and Resuscitation Service, University Clinical Hospital of Valladolid, Valladolid, Spain; ^9^ Anesthesiology and Resuscitation Service, University Clinical Hospital of Salamanca, Salamanca, Spain; ^10^ Institute of Applied Ophthalmobiology (IOBA), University of Valladolid, Valladolid, Spain; ^11^ Biomedical Research Networking Centre in Bioengineering, Biomaterials, and Nanomedicine (CIBERBBN), Carlos III National Institute of Health, Madrid, Spain; ^12^ Vascular Surgery Service, University Clinical Hospital of Valladolid, Valladolid, Spain; ^13^ Mucosal Immunology Laboratory, Institute of Biology and Molecular Genetics (IBGM), University of Valladolid-Spanish National Research Council, Valladolid, Spain; ^14^ Biomedical Research Networking Centre in Hepatic and Digestive Diseases (CIBEREHD), Carlos III National Institute of Health, Madrid, Spain

**Keywords:** COVID-19, ABO blood groups, cytokines, mortality, hepatocyte growth factor

## Abstract

Severe status of coronavirus disease 2019 (COVID-19) is extremely associated to cytokine release. Moreover, it has been suggested that blood group is also associated with the prevalence and severity of this disease. However, the relationship between the cytokine profile and blood group remains unclear in COVID-19 patients. In this sense, we prospectively recruited 108 COVID-19 patients between March and April 2020 and divided according to ABO blood group. For the analysis of 45 cytokines, plasma samples were collected in the time of admission to hospital ward or intensive care unit and at the sixth day after hospital admission. The results show that there was a risk of more than two times lower of mechanical ventilation or death in patients with blood group O (log rank: *p* = 0.042). At first time, all statistically significant cytokine levels, except from hepatocyte growth factor, were higher in O blood group patients meanwhile the second time showed a significant drop, between 20% and 40%. In contrast, A/B/AB group presented a maintenance of cytokine levels during time. Hepatocyte growth factor showed a significant association with intubation or mortality risk in non-O blood group patients (OR: 4.229, 95% CI (2.064–8.665), *p* < 0.001) and also was the only one bad prognosis biomarker in O blood group patients (OR: 8.852, 95% CI (1.540–50.878), *p* = 0.015). Therefore, higher cytokine levels in O blood group are associated with a better outcome than A/B/AB group in COVID-19 patients.

## 1. Introduction

The novel severe acute respiratory syndrome coronavirus 2 (SARS-CoV-2) (1, 2) is responsible for coronavirus disease 2019 (COVID-19) and is currently one of the worst pandemics after the 1918 Spanish influenza virus (3). Although most of the cases are asymptomatic or shows mild symptoms, more than 75% of hospitalized patients need supplemental oxygen, with 20% of them requiring intensive care unit (ICU) admission (4).

Severe infection is associated with age, sex, and comorbidities. It has also been suggested the role of ABO blood group could play in COVID-19 (5–8). In fact, recent studies reported blood group O patients associate lower prevalence and severity of symptoms, while blood group A or AB present an increased risk of requiring mechanical ventilation, renal replacement therapies, and prolonged ICU admission (9, 10).

SARS-CoV-2 binds to angiotensin-converting enzyme 2 (ACE2) of target cells (11, 12), so it is reasonable to consider blood group as a susceptibility marker in COVID-19 patients. Moreover, previous studies demonstrated that SARS-CoV-1 particles can be glycosylated by the A variant of the ABO glycosyltransferases (13). On the other hand, it has been reported that anti-A antibody can also bind to ACE2. Although it has not been studied, it is likely that SARS-CoV-2 competes against anti-A, and maybe with anti-B, antibodies to bind to ACE2 from host cells. These antibodies are present on mucosal surfaces in some individuals lacking the corresponding ABO blood group, which could explain the relative protection of blood group O individuals (14).

Severe and critical patients show lymphopenia (15) or increased levels of serum C-reactive protein (CRP), hypoalbuminemia, alanine aminotransferase, lactate dehydrogenase, ferritin, and/or D-dimer (16, 17). In addition, these patients display increased levels of serum proinflammatory cytokines such as IL-1β, IL-6, IL-12, IFN-γ, IP10, or MCP1 (18, 19) which are related to group 1 T helper cell responses (Th1). Moreover, more severe patients (including those who require ICU admission) display higher plasma levels of G-CSF, IP10, MCP1, MIP1A, and TNF-α, suggesting an association with the degree of severity (20–22). Consequently, if ABO blood group effectively influences the severity or mortality of COVID-19 patients, it would be expected that different behaviors in cytokine response would be identified. Nevertheless, and to our knowledge, only one study has considered the cytokine profile of COVID-19 patients in the context of ABO blood group and only the expression levels of four proinflammatory cytokines (IL-1β, IL-6, IL-10, and TNF-α) were studied (9).

Therefore, and building on this gap in the knowledge, we aimed to prospectively perform a 45-cytokine array in plasma samples, at two different moments during the hospital stay, from newly diagnosed COVID-19 patients who were stratified based on their blood group. Thus, the main goal was to provide further insight into the possible protective mechanisms elicited by blood group O through cytokine profile and its evolution and at the same time, to find possible bad prognosis biomarkers through ABO implications in COVID-19 disease.

## 2. Materials and Methods

### 2.1 Patient Selection

A representative cohort of hospitalized COVID-19 patients was recruited. A total of 108 adult patients, aged over 18 years, who were diagnosed with COVID-19 and admitted to the Hospital Clínico Universitario de Valladolid (Spain) were prospectively and consecutively recruited between 24th March and 11th April 2020. For inclusion of the patients in the present study, confirmation of SARS-CoV-2 infection using the polymerase chain reaction (PCR) on a nasopharyngeal sample was necessary. In addition, 28 patients admitted to the hospital in the same time period for elective major surgery with a negative PCR result for SARS-CoV-2 infection were included as control for normalization of the results. Patients with any other infection at the time of COVID-19 diagnosis or chronic terminal illness were not included. Demographic, clinical, and analytical data were also obtained from each patient.

### 2.2 Biological Samples

We prospectively obtained plasma samples from each patient at two different moments during the hospital stay:

- Admission to hospital ward or ICU (108 samples).
- Sixth day after hospital admission (86 samples).

All samples were collected at 9 a.m. in order to prevent circadian cycle variations. Blood was collected in 3.2% sodium citrate tubes and centrifuged at 2,000×*g* for 20 min at room temperature. The resulting plasma was aliquoted and directly frozen at −80°C until use.

### 2.3 Blood Group

Blood group determination was performed using the samples in a fully automated analyzer (Erytra automated system for blood typing) using the DG gel card technology. Patients were divided in two groups: (i) blood group O and (ii) blood group non-O (A/B/AB).

### 2.4 Cytokine and Chemokine Analysis

The quantification of soluble mediators was performed using the previous plasma aliquots. Cytokines were measured in duplicate for each patient using a MAGPIX system (Luminex). Forty-five protein targets were analyzed with the Cytokine/Chemokine/Growth Factor 45-Plex Human ProcartaPlex™ Panel 1 (Invitrogen, Waltham, MA, USA) following the manufacturer’s guidelines and recommendations. Cytokines and chemokines included in the panel were brain-derived neurotrophic factor (BDNF), epidermal growth factor (EGF), Eotaxin (also known as CCL11), FGF-2, GM-CSF, GRO-α (CXCL1), HGF, IFN-α, IFN-γ, IL-1α, IL-1β, IL-10, IL-12 p70, IL-13, IL-15, IL-17a (CTLA-18), IL-18, IL-1RA, IL-2, IL-21, IL-22, IL-23, IL-27, IL-31, IL-4, IL-5, IL-6, IL-7, IL-8 (also known as CXCL8), IL-9, IP-1 beta (CCL4), IP-10 (CXCL10), LIF, MCP-1 (CCL2), MIP-1α (CCL3), NGF-β, PDGF-BB, PIGF-1, RANTES (CCL5), SCF, SDF-1α, TNF-α, TNF-β, VEGF-A, and VEGF-D.

### 2.5 Ethics

The study was approved by the Clinical Ethics Committee (CEIm) of the hospital, and approval was obtained from all study participants (cod: PI 20-1717). This study followed the code of ethics of the World Medical Association (Declaration of Helsinki).

### 2.6 Statistical Analysis

Descriptive statistics were used to summarize demographic data, clinical characteristics and analytical data. Categorical variables were expressed as the total number and percentage [*n* (%)], and significance was assessed by the Chi-square test. Continuous variables were represented by the median and interquartile range [median (IQR)], and significance was tested using Mann Whitney *U*.

To assess whether the ABO blood group was related to severity and a different cytokine profile (see ***Results***), patients were divided into two groups: group O and group non-O (A/B/AB). In addition, both groups were in turn subdivided according to the final outcome (intubation or death).

Cytokines need to reach a level of at least 20% in every sample in order to ensure robust results. The nondetects and data analysis (NADA) R package (23) was used to perform a regression to impute low values after checking that the data follow a log-normal distribution. Molecules outside of those conditions were not statistically analyzed any further. The logarithmic base 2 scale was employed in the cytokine expression data. The different cytokine profile, in each moment, was again evaluated using the median and interquartile range [median (IQR)], and significance was tested using Mann-Whitney *U*.

The strength of each cytokine to define the different profiles according to blood group was evaluated in several univariate regression models adjusted for the same age and gender. The main variable was blood group, which is a categorical. These models require compliance with the proportional odds assumption. To confirm this, the proportional odds model was compared with a multinomial logistic regression model through the likelihood ratio test. However, in none of the cases was it possible to assume this hypothesis, so multinomial models were fitted. In the same way, in each ABO blood group, the relationship between cytokines and intubation or death risk was also evaluated.

To evaluate the cytokine evolution over the days, an analysis included variations in cytokines levels, expressed in percentage, comparing first and second moments. It was also described according to main variable (Blood group O).

The univariable regression model was performed to associate the intubation or mortality risk and hepatocyte growth factor (HGF) levels in the group of O blood group patients. The model was internally validated with the leave-one-out-cross-validation (LOOCV) procedure and the receiver operating characteristic (ROC) curve analysis.

The cumulative event rate based on death or requirements of mechanical ventilation was performed using the Kaplan-Meier method by comparing blood group A/B/AB and blood group O. Cumulative incidence curves were determined with the log-rank test. The stratified Cox proportional-hazards model was used to estimate the hazard ratio (for blood group A/B/AB as referred to blood group O) and 95% confidence interval. Data on patients who survived and did not require mechanical ventilation before day 28 were censored at the last follow-up date or on day 28, whichever occurred first.

Statistical analysis was performed by a PhD-licensed statistician using the R statistical package version 3.1.1 R Core Team and statistical package IBM SPSS Statistics software (SPSS) version 25. Statistical significance was set at *p* ≤ 0.05.

## 3. Results

### 3.1 Admission to Hospital Ward or Intensive Care Unit

#### 3.1.1 Demographics

Our cohort included 108 patients with a median age of 67 years and was mostly male (63.26%). The percentage distribution of ABO blood group corresponds to 54.6%, 9.3%, 3.7%, and 32.4% for A, B, AB, and O groups, respectively. In our analysis, we established two different groups: Blood group O (*n* = 35, 32.4%) and blood group no-O or A/B/AB (*n* = 73, 67.6%). The patients’ clinical and analytical profiles are shown in **Table 1**. Patients did not differ regarding age, gender, or comorbidities. In both groups, the most common comorbidities were hypertension (50.7% and 37.1%), diabetes (18.2% and 14.3%), and COPD (16.45 and 11.4%). Blood group O patients had higher lymphocyte (*p* = 0.057) and lower total bilirubin (*p* = 0.009) plasma levels than the A/B/AB group. Moreover, the requirement for mechanical ventilation in ICUs was higher in the A/B/AB group (*p* = 0.019), who also had an increased risk of mortality (although borderline, *p* = 0.067) and a longer period of hospitalization (*p* = 0.053).

**Table 1:** Clinical characteristics of the patients.

	Group A/B/AB (*N* = 73)	Group O (*N* = 35)	*p*-Value
Characteristics			
Age [median (IQR)]	71.5 (68)	64.50 (66)	0.226
Male [*n* (%)]	33 (47.1)	14 (41.2)	0.566
Comorbidities [*n* (%)]			
Smoking	5 (6.8)	4 (11.4)	0.467
Coronary disease	7 (9.5)	3 (8.6)	0.864
Atrial fibrillation	8 (11.0)	4 (11.4)	0.942
Diabetes	14 (18.2)	5 (14.3)	0.646
Neurological disease	1 (1.4)	1 (2.9)	0.545
Stroke	2 (2.8)	1 (2.9)	0.545
Hypertension	37 (50.7)	13 (37.1)	0.187
Liver disease	2 (2.7)	0 (0.0)	0.323
Obesity	7 (9.6)	3 (8.6)	0.864
COPD	12 (16.4)	4 (11.4)	0.549
Kidney disease	3 (4.1)	0 (0.0)	0.549
Laboratory [median (IQR)]			
Glycemia (mg/dl)	145 (105)	148 (87.5)	0.815
Creatinine (mg/dl)	0.9 (0.6)	0.8 (0.3)	0.105
Total bilirubin (mg/dl)	1.0 (1.1)	0.7 (0.3)	**0.011**
Leukocytes (×10^9^/L)	7.3 (4.5)	7.1 (4.9)	0.841
Lymphocytes (×10^9^/L)	0.7 (0.6)	0.8 (1.0)	**0.057**
Neutrophil (×10^9^/L)	5.7 (4.4)	5.7 (5.1)	0.939
Procalcitonin (ng/ml)	0.1 (0.3)	0.1 (0.2)	0.415
Platelet (×10^9^/L)	273 (148)	301.5 (167)	0.343
CRP (mg/L)	42.0 (61)	34.5 (30)	0.348
Ferritin (μg/L)	1000 (1247)	920.0 (927)	0.220
D-Dimer (mg/L)	872.5 (1219)	770 (1003)	0.690
Lactate (mmol/L)	2.2 (1.0)	2.9 (0.9)	0.672
Hospital meters			
Invasive mechanical ventilation [*n* (%)]	26 (35.6)	6 (17.1)	**0.019**
Length of hospital stay [days, median (IQR)]	26.5 (39)	33 (25)	**0.053**
Length of ICU stay [days, median (IQR)]	19 (16)	19 (21)	0.947
Mortality [*n* (%)]			
28-day mortality	17 (23.3)	3 (8.6)	**0.065**

Continuous variables are represented as [median (interquartile range, IQR)]; categorical variables are represented as [n, (%)]. COPD, chronic obstructive pulmonary disease; CRP, C-reactive protein; ICU, intensive care unit.

A -value p <= 0.05 was considered to indicate significant differences (bold values).

#### 3.1.2 ABO Blood Group and Severity or Mortality Risk

**Figure 1:**
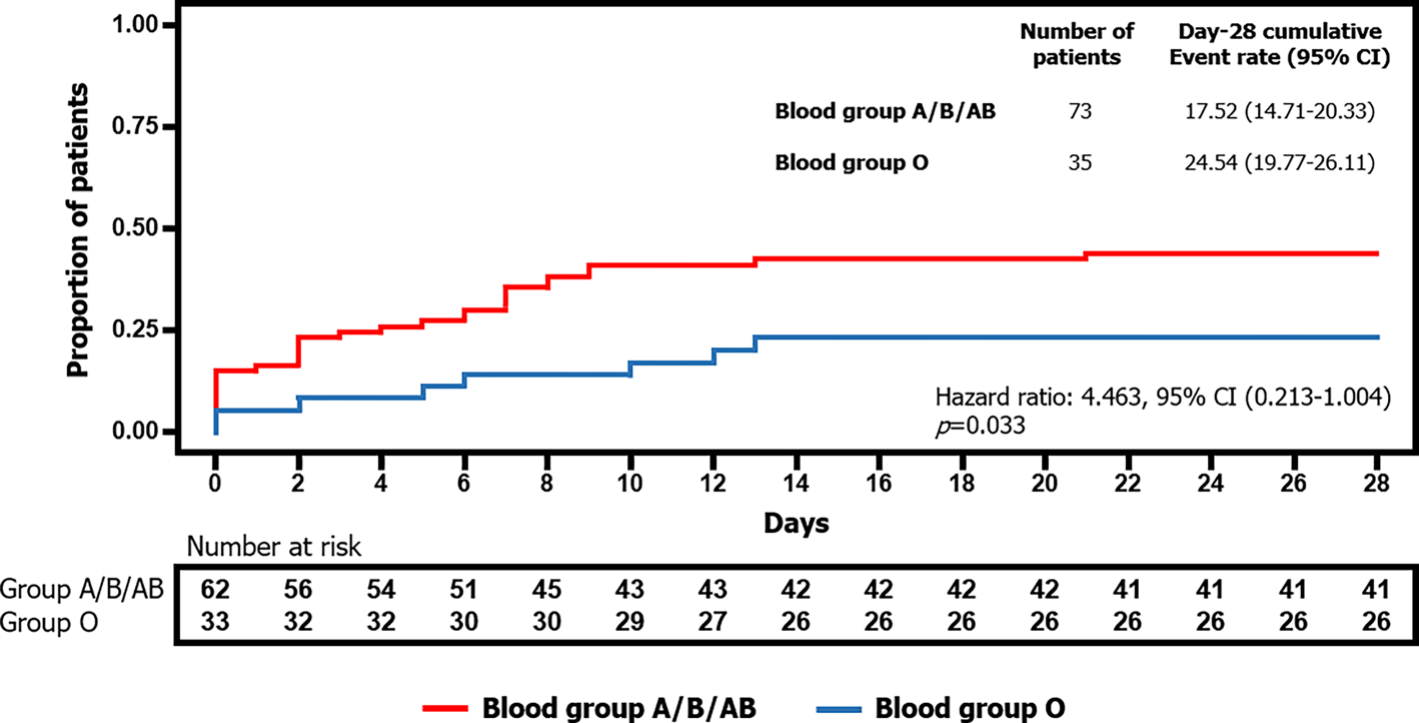
Differences in time to mechanical ventilation or death by day 28 depending on blood group A/B/AB or blood group O.

The Kaplan-Meier curve (**Figure 1**) revealed how the cumulative percentage of patients with a critical prognosis (i.e., those who required mechanical ventilation or died by day 28) was significantly lower in the blood group O (log rank: *p* = 0.042). The stratified Cox proportional-hazards model also showed statistical significance. Hence, blood group O was associated with 2.16 (1/0.463) times lower probability of mechanical ventilation or death (hazard ratio: 0.463, 95% CI (0.213–1.004), *p* = 0.050).

#### 3.1.3 Cytokine Profile According to ABO Blood Groups

**Figure 2:**
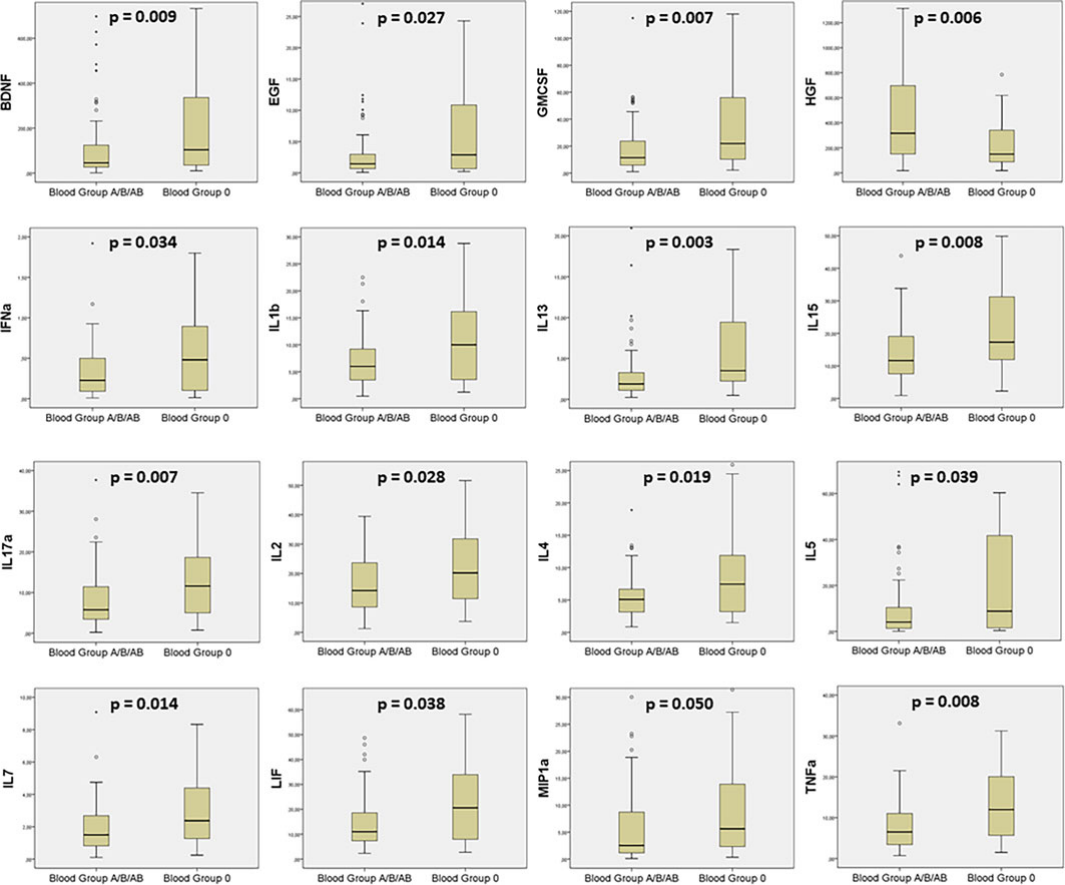
Boxplots showing the statistically significant cytokines comparing blood group A/B/AB and blood group O patients.

A total of eight cytokines did not reach the minimum detection rate of 20% required to assume log-normal quantiles for samples. Hence, FGF-2, IL-12, IL-21, IL-23, IL-31, IL-9, NGF-β, and TNF-β were excluded from the analysis. Assessment of the remaining 37 cytokines (**Supplementary Table S1A**) revealed that 16 of them were statistically different between the two groups (**Figure 2**). BDNF, EGF, GMCSF, IFN-α, IL-1β, IL-13, IL-15, IL-17α, IL-2, IL-4, IL-5, IL-7, LIF, MIP1a, and TNF-α were higher in blood group O, while HGF was the only one underexpressed in this group. Based on a likelihood ratio test, the most plausible model in all cases was the multinomial one. Therefore, we performed individual gender- and age-adjusted multinomial models for each cytokine, which concluded the same results (**Supplementary Figure S1**).

### 3.2 Sixth Day After Hospital Admission

#### 3.2.1 Demographics

After 6 days in ward or ICUs, 22 patients were censored for the following cytokine analyses. On the one hand, 11 patients with O blood group were discharged from hospital, without any deaths. On the other hand, there were eight patients discharged and three deaths in A/B/AB blood groups. Therefore, this time, total number of patients was 86, including 24 patients with blood group O (27.9%) and 62 patients with blood group no-O or A/B/AB (72.1%).

#### 3.2.2 Evolution on the Cytokine Profile

**Figure 3:**
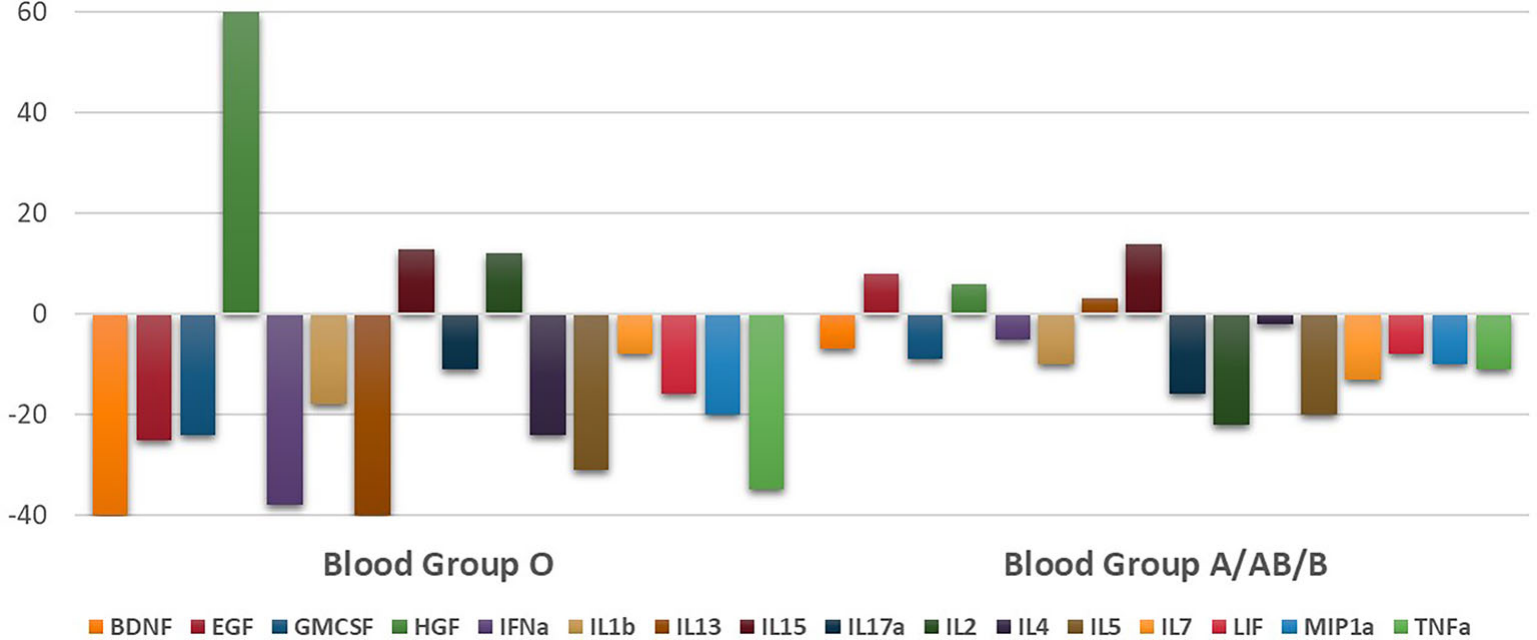
Evolution of the level of cytokines represented by the percentage change of each one after 6 days of hospital admission.

Comparing with first cytokine determination, the sixth day of admission the plasma levels of only four cytokines showed statistically significant differences between ABO groups (**Supplementary Table S1B**). IL-1β, IL-15, IL-17α, and IL-2 were the only ones that remained overexpressed in O blood groups patients. Cytokine evolution over the days showed a significant drop in levels of the majority of cytokines in O blood group (**Figure 3**). In fact, nine cytokines presented between 20% and 40% lower levels than first determination in O blood group. Conversely, only three cytokines in this blood group showed increased levels and HGF associated the widest difference, 60% of increase. Nevertheless, in A/B/AB blood group, cytokine levels showed stabilization at this time, with few percentage variations comparing with first determination. There were only two cytokines that could reach a drop around 20%. In this subgroup, the maintenance of the levels was evident (**Figure 3**). Full material is shown in **Supplementary Table S2**.

#### 3.2.3 Relationship Between Cytokines and Outcome Depending on Each ABO Blood Group

The stratification according to ABO blood groups let us know in a direct way the association between each cytokine level and the intubation or death risk (bad outcome) at first and second moments (**Supplementary Tables S3A–D**).

**Figure 4:**
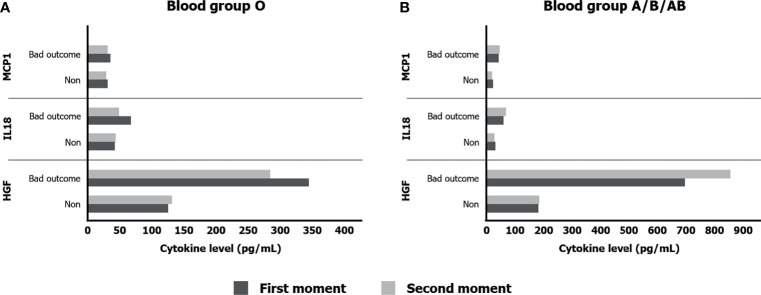
Significant cytokines levels according to bad outcome (intubation or death) in O blood group **(A)** and their expression in A/B/AB blood group patients **(B)**.

In O blood group, only HGF (*p* = 0.015) and PDFGBB (*p* = 0.028) were related to bad outcome at first determination, meanwhile some cytokines appeared to be in second time. We should take into consideration the scant number of patients; above all in sixth day of hospital stay (24 patients). In A/B/AB blood group, three cytokines found association with disease severity in both moments. First one, it was again HGF (*p* < 0.001 in both times). In addition, IL-18 (*p* = 0.048 and *p* = 0.036) and MCP1 (*p* < 0.001 and *p* = 0.002) were also significant. Comparing those cytokines in non-O blood group patients (**Figure 4A**), HGF levels were much more elevated in intubated or death patients (up four times). This tendency in HGF was newly evidenced in O blood group, contrasting with the other two cytokines (**Figure 4B**).

#### 3.2.4 HGF and Its Relationship With ABO Blood Groups and Severity

**Figure 5:**
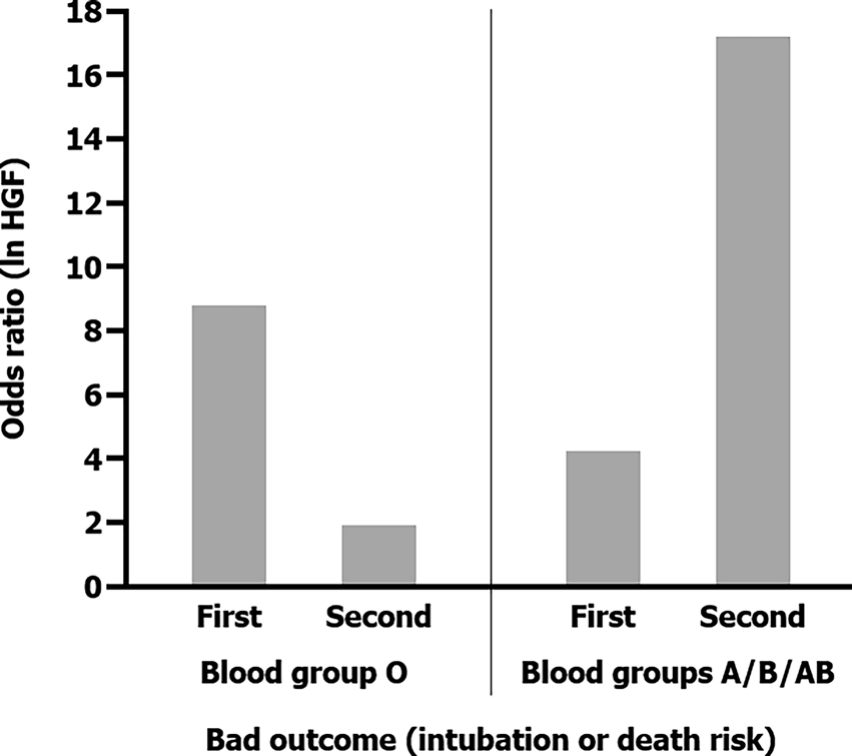
Different odds ratios obtained through four gender- and age-adjusted odd ratio univariable models which associated bad outcome (intubation or death) and HGF levels according to both ABO blood groups and also hospital stay moments.

HGF was the only one cytokine that showed significant higher levels in A/B/AB patients, the ones who associated worse prognosis. In the evolution analyses, HGF increased its levels in both blood groups. Despite the change was clearer in O blood group patients (60%, 149 to 240 pg/ml), levels were still lower than A/B/AB patients (6%, 316 to 337 pg/ml). Building on that, a gender- and age-adjusted odds ratio univariable model found association between higher HGF levels and intubation or mortality risk in O blood group patients [OR: 8.852, 95% CI (1.540–50.878), *p* = 0.015] and also in A/B/AB one [OR: 4.229, 95% CI (2.064–8.665), *p* < 0.001] after hospital admission. The LOOCV procedure was used in the internal validation of this model. It revealed an area under the curve (AUC) of 0.871 and 0.835, respectively, which were significantly greater than 0.5. Hence, the persistent elevated HGF level in non-O group patients was even more associated with bad prognosis, reaching 17 times more intubation or death risk after 6 days of hospital stay [OR: 17.268, 95% CI (4.287–69.550), *p* < 0.001] with 0.934 of AUC (**Figure 5**).

## 4. Discussion

The implications of ABO blood groups into the pathophysiology of COVID-19 would be explained through a cytokine profile and its evolution during hospital stay, providing the comprehension of molecular mechanism and the finding of new prognosis biomarkers. In this sense, our prospective clinical study confirmed a better prognosis in O blood group patients, associating lower risk of mechanical ventilation or death (2.16 times; log rank: *p* = 0.042, hazard ratio: 0.463, 95% CI (0.213–1.004), *p* = 0.050). At first moment, all statistically significant cytokine levels, except from HGF, were higher in O blood group patients meanwhile the second moment showed a significant drop, between 20% and 40%, into the majority of cytokine levels in patients with O blood group. Last but not least, HGF was the only one cytokine that increased its levels in both blood groups during hospital stay. A significant association with intubation or mortality risk was demonstrated, along with IL-18 and MCP1, in A/B/AB blood group (OR: 4.229, 95% CI (2.064–8.665), *p* < 0.001), and it was the only one cytokine that associated bad outcome in O blood group (OR: 8.852, 95% CI (1.540–50.878), *p* = 0.015).

Since the beginning of the SARS-CoV-2 pandemic, several comorbidities were described as early risk factors (22). In 1901, Karl Landsteiner discovered the ABO blood group system, which has been linked to various infectious diseases such as *Helicobacter pylori* (24), *Plasmodium falciparum* (25), norovirus (26), hepatitis B virus (27), SARS-CoV (13), and MERS-CoV (28). Recently, new studies have demonstrated the relationship between blood group O and a lower mortality and severity in COVID-19 patients (14, 29–31). Our results confirm these observations and expand them. Indeed, the results for the general population covered by our hospital are 42%, 8%, 3%, and 47% for blood groups A, B, AB, and O, respectively (32). Nevertheless, in our cohort of patients admitted to hospital, we found an increase in the number of patients with blood group A (54.6%) and a significant decrease in patients with blood group O (32.4%). Therefore, blood group O was not only associated with a lower risk of mortality or mechanical ventilation but also with the need for hospital admission. Indeed, and although the impact of blood group on COVID-19 outcome seems clear, few studies have performed robust investigations (9, 10, 33) not only showing the lower risk of intubation or death in blood group O patients but also trying to understand the protective mechanisms and serum mediators involved.

**Figure 6:**
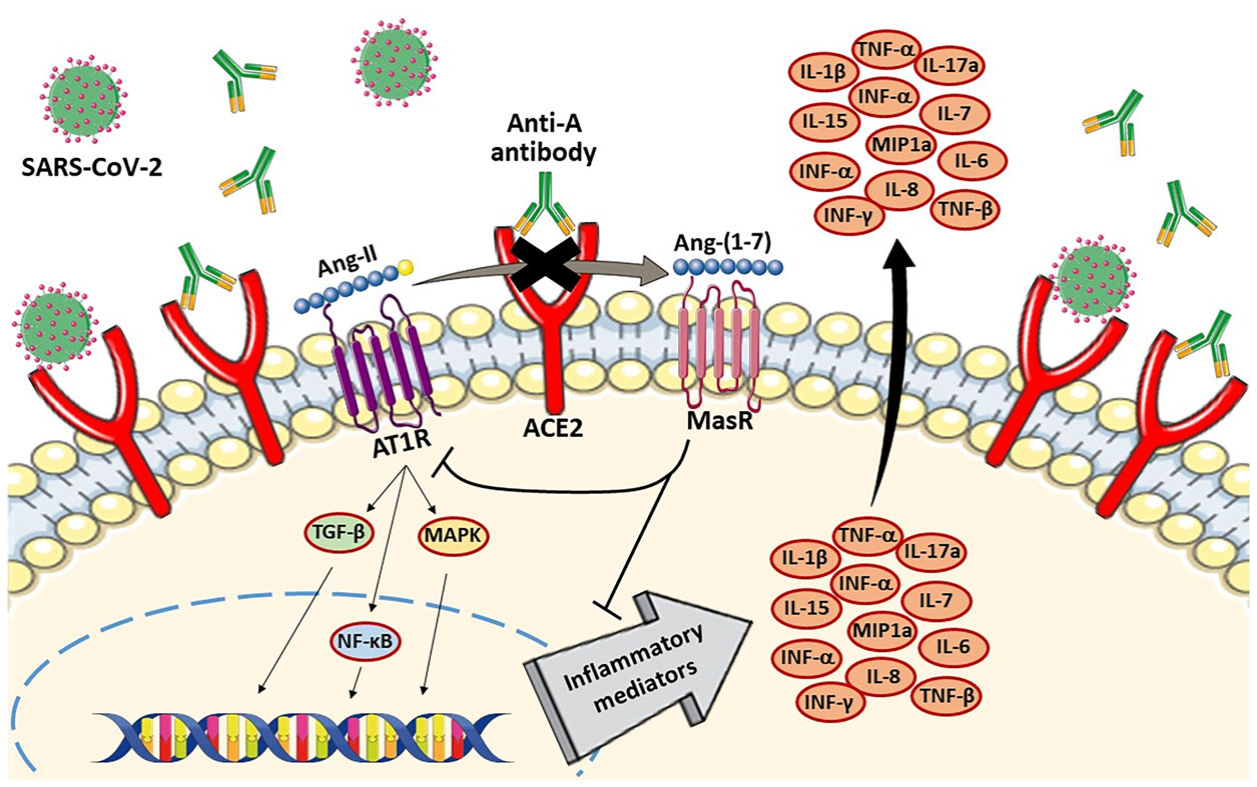
Competitive inhibition of ACE2 by both anti-A antibodies and SARS-CoV-2 in blood group O patients. The SARS-CoV-2 and anti-A antibodies induced ACE2 downregulation. Ang-II can activate NF-κB pathway in AT1R-mediated inflammatory response, leading to the increased production of multiple inflammatory cytokines. Therefore, SARS-CoV2 had more difficulties to use ACE2 as the cellular entry receptor because of anti-A antibodies.

The first molecular mechanism lies directly to understand the relationship between ACE2, SARS-CoV-2, and ABO blood groups (anti-A and anti-B antibodies), and in a second step, to describe the implications of those associations with different intracellular signaling pathways and the repercussions in cytokine release. SARS-CoV-2 uses ACE2 in group II pneumocytes of the lung alveoli as the cellular entry receptor. Binding of the virus to ACE2 triggers ACE2 downregulation, along with increased angiotensin II (Ang-II) and decreased angiotensin-(1-7) levels (34). Ang-II can activate the nuclear factor kappa B (NF-κB) pathway, leading to the increased production of multiple inflammatory cytokines such as TNF-α or IL-1β (35). Therefore, the imbalance of ACE2/ACE and the Ang-II/AT1R axis could explain the increase in cytokine levels in COVID-19 patients, as well as the associated lung damage. In a similar way, it is known that anti-A antibodies existing in blood group O patients block the ACE2 receptor (36), suggesting that the same could happen with anti-B antibodies. This hypothesis was confirmed in studies of patients with SARS-CoV infection, showing how anti-A antibodies specifically inhibited the SARS-CoV S protein/ACE2-dependent adhesion (13). Both SARS-CoV-2 and anti-A and anti-B antibodies allow the strong competitive inhibition of ACE2 in blood group O patients. This ACE2 downregulation associates high Ang-II levels, which allows the production of inflammatory cytokines, and, at the same time, a lower infectious capacity by SARS-CoV-2 in blood group O patients (**Figure 6**). Therefore, this relationship between ACE2, SARS-CoV-2, and anti-A and anti-B antibodies could explain the higher cytokine levels in O blood group patients, as well as their better prognosis. One of the few studies carried out to describe the inflammatory profile with several serum mediators according to ABO blood group was performed by Hoiland et al. However, they only studied four cytokines without obtaining clear evidence or statistically significant results.

Apart from this first mechanism involved in the pathophysiology of the virus, which was previously reported (35), our results provide another explanation for the better prognosis of O blood group patients. In this sense, an early and effective activation of the immune response plays a capital role. Our cytokine analyses along hospital stay and their differences between ABO blood groups and outcome let us assume that importance. The existence of a higher activation status of the immune system—displayed by higher levels of 15 cytokines at hospital admission—is of particular relevance given that early activation of the immune response is associated with rapid viral clearance and mild disease in COVID-19 patients (37). Indeed, patients with a worse prognosis display a deficient primary innate immune response (38, 39) as also confirmed in animal models (40). In this way, our cytokine evolution analysis proved a significant drop, between 20% and 40%, in the majority of cytokines levels in O blood group and, by cons, minimum percentage variations and a maintenance of levels in A/B/AB blood group. Therefore, our results could be a reason in favor of the rapid activation of the immune response in patients with blood group O, the key for a more rapid viral clearance and, therefore, a better disease prognosis.

According to the association between the HGF and higher risk of intubation or dead outcome, studies support this theory (41, 42). HGF is produced by stromal cells and is required for self-repair after organ damage in the liver, lung, or kidney. It inhibits inflammation by interfering NF-κB pathway (43). Insufficient HGF is related to organ failure and even, in animal models, anti-HGF antibodies increased tissue destruction (43). Moreover, in our research, HGF was the only one cytokine that showed significant higher levels in A/B/AB patients, the ones who associated worse prognosis. In fact, in non-O group there were only three cytokines (HGF, MCP1, and IL-18) with persistent higher levels during hospital stay, being related to bad outcome with significant results in regression analyses. In COVID-19, this may represent an indirect expression of lung damage as a consequence of inflammation (41–43), much more in the A/B/AB blood group. Cytokine evolution analysis showed increase percentage levels clearer in O blood group patients (60%, 149 to 240 pg/ml), although levels were still lower than A/B/AB patients (6%, 316 to 337 pg/ml). For this reason, through this cytokine profile in ABO blood groups, we could find HGF as a bad prognosis biomarker in all COVID-19 patients (both ABO groups), growing up to 17 times more intubation or death risk in non-O patients with persistently higher levels. In the group with worse outcome, A/B/AB group, the existence of other two proinflammatory cytokines (MCP1 and IL-18) with a maintenance of levels after 6 days of hospital stay in bad outcome patients supports the immune system dysregulation and the permanent inflammatory status that intubated or death patients associated in non-O blood group. By cons, in the blood group O patients, levels of all cytokines, except from HGF, are lowered in the second moment due to an optimal immune response and the competitive inhibition of ACE2. In the O blood group, our study only found high levels of HGF as the ones more clearly responsible for bad outcome.

Nevertheless, we are aware of the limitations of our study, including the monocentric cohort, focus on hospitalized patients and the performance of an indirect study based on cytokine profile and the absence of specific cellular one by, for example, flow cytometry. Future investigations should therefore expand these observations and confirm not just the lower viral load of patients with blood group O but also to perform functional studies to identify the specific mechanisms by which these patients are more protected.

## Conclusions

A prospective study at two different moments during the hospital stay in COVID-19 patients confirmed both lower rates of hospital admission and a lower risk of intubation or death in O blood group. Better prognosis in the O blood group was associated with higher levels in all statistically significant cytokines, except from HGF, at first moment, and a consequent significant drop after 6 days of hospital stay. Those findings would also explain an early and effective activation of the immune response in the O blood group, associating a rapid viral clearance of the viral infection as well as the important relationship between ACE2, SARS-CoV-2, and anti-A and anti-B antibodies into the pathophysiology of COVID-19. Moreover, HGF was proven as a bad prognosis biomarker through this ABO cytokine profile study.

## Data Availability Statement

The raw data supporting the conclusions of this article will be made available by the authors, without undue reservation.

## Ethics Statement

The studies involving human participants were reviewed and approved by Hospital’s Clinical Ethics Committee of Valladolid (CEIm). The patients/participants provided their written informed consent to participate in this study.

## Author Contributions

Literature search: ÁT-V and ET. Study design: ÁT-V, ET, HG-B, and PM-P. Figures: IFe, IFu, and SP-G. Data collection: ÁT-V, EG-S, LuR, MJ, AR, and MV. Data analysis: MM-M and ÁT-V. Data interpretation: MH-R, ÓG-G, FÁ, MP-P, CD, LoR, IC-F, and MM-F. Writing: ÁT-V, ET, and DB. Supervision and visualization: ET, DB, and PM-P. All authors contributed to the article and approved the submitted version.

## Funding

The present study was supported by the Carlos III Health Institute (Spain) (Grant COV20/00491) and the Junta de Castilla y León (Spain) (Grant 18IGOF).

## Conflict of Interest

The authors declare that the research was conducted in the absence of any commercial or financial relationships that could be construed as a potential conflict of interest.

## Publisher’s Note

All claims expressed in this article are solely those of the authors and do not necessarily represent those of their affiliated organizations, or those of the publisher, the editors and the reviewers. Any product that may be evaluated in this article, or claim that may be made by its manufacturer, is not guaranteed or endorsed by the publisher.

## Supplementary Material

Supplementary Figure 1 Individual logistic regression models on each cytokine. OR, odds ratio; CI, confidence interval; Cit, cytokine. OR was expressed in logarithmic base 2.
