# Supplementary figures and images for: Can the Cytokine Profile According to ABO Blood Groups Be Related to Worse Outcome in COVID-19 Patients? Yes, They Can
Source: Front Immunol. 2021 Oct 13;12:726283. doi: 10.3389/fimmu.2021.726283 (PMC8548690; doi:10.3389/fimmu.2021.726283)

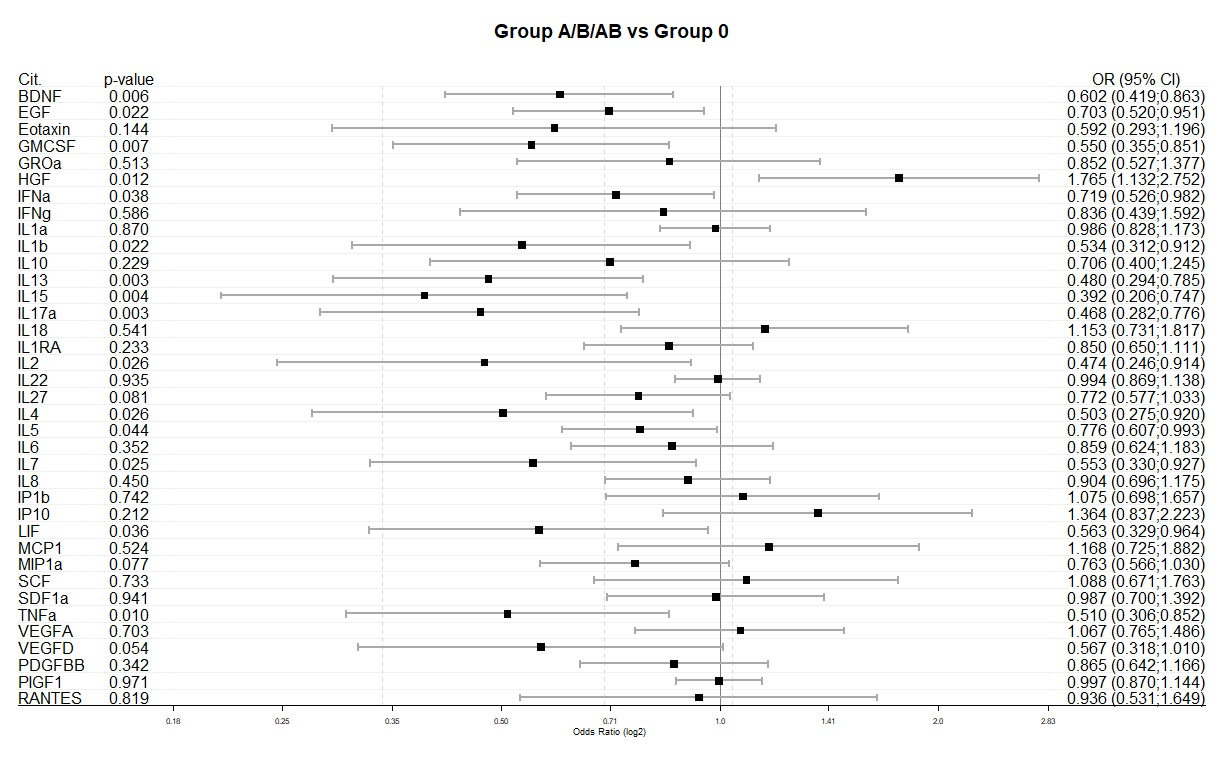

Supplement: Supplementary Figure 1 — Individual logistic regression models on each cytokine. OR, odds ratio; CI, confidence interval; Cit, cytokine. OR was expressed in logarithmic base 2. [file Image_1.tif]
