# Supplementary material for: Can the Cytokine Profile According to ABO Blood Groups Be Related to Worse Outcome in COVID-19 Patients? Yes, They Can
Source: Front Immunol. 2021 Oct 13;12:726283. doi: 10.3389/fimmu.2021.726283 (PMC8548690; doi:10.3389/fimmu.2021.726283)
Supplement: Supplementary file 2 [file Table_1.docx]

| **First (a)** | **Group O**  **(N=35)** | | **Group A/B/AB**  **(N=73)** | | ***p*** |
| --- | --- | --- | --- | --- | --- |
|  | **Median** | **IQR** | **Median** | **IQR** |  |
| BDNF | 103.80 | 300.95 | 45.75 | 97.70 | 0.009 |
| EGF | 2.88 | 10.17 | 1.45 | 2.31 | 0.027 |
| Eotaxin | 15.98 | 8.82 | 12.45 | 9.59 | 0.123 |
| GMCSF | 21.98 | 45.73 | 11.46 | 17.83 | 0.007 |
| GROa | 3.03 | 2.64 | 2.25 | 2.03 | 0.403 |
| HGF | 149.00 | 253.25 | 316.50 | 546.25 | 0.006 |
| IFNa | 0.48 | 0.79 | 0.23 | 0.41 | 0.034 |
| IFNg | 9.96 | 7.04 | 8.59 | 6.74 | 0.356 |
| IL1a | 2.55 | 5.25 | 2.47 | 7.16 | 0.713 |
| IL1b | 10.01 | 12.61 | 5.99 | 5.75 | 0.014 |
| IL10 | 1.63 | 1.85 | 1.35 | 0.88 | 0.172 |
| IL13 | 3.51 | 7.20 | 1.89 | 2.17 | 0.003 |
| IL15 | 17.30 | 19.30 | 11.65 | 11.53 | 0.008 |
| IL17a | 11.60 | 13.62 | 5.77 | 8.00 | 0.007 |
| IL18 | 48.15 | 52.50 | 43.20 | 65.10 | 0.865 |
| IL1RA | 866.00 | 1027.67 | 455.50 | 977.50 | 0.102 |
| IL2 | 20.23 | 20.34 | 14.20 | 15.02 | 0.028 |
| IL22 | 7.09 | 14.17 | 2.20 | 13.76 | 0.487 |
| IL27 | 27.54 | 100.56 | 15.29 | 35.56 | 0.076 |
| IL4 | 7.44 | 8.69 | 5.09 | 3.53 | 0.019 |
| IL5 | 8.82 | 40.10 | 4.10 | 9.08 | 0.039 |
| IL6 | 11.33 | 27.75 | 8.32 | 13.14 | 0.125 |
| IL7 | 2.38 | 3.13 | 1.50 | 1.86 | 0.014 |
| IL8 | 2.34 | 5.67 | 1.72 | 4.05 | 0.434 |
| IP1b | 49.25 | 47.90 | 51.10 | 43.10 | 0.664 |
| IP10 | 20.38 | 22.52 | 28.75 | 32.70 | 0.103 |
| LIF | 20.60 | 25.98 | 11.01 | 11.23 | 0.038 |
| MCP1 | 32.28 | 35.28 | 32.05 | 32.90 | 0.708 |
| MIP1a | 5.62 | 11.58 | 2.53 | 7.57 | 0.050 |
| PDGFBB | 517.75 | 882.50 | 414.00 | 1051.85 | 0.311 |
| PIGF1 | 4.74 | 45.32 | 4.76 | 69.74 | 0.723 |
| RANTES | 27.23 | 25.00 | 26.45 | 23.85 | 0.434 |
| SCF | 7.35 | 6.06 | 6.88 | 7.46 | 0.875 |
| SDF1a | 654.25 | 554.50 | 689.67 | 656.50 | 0.857 |
| TNFa | 11.98 | 14.39 | 6.52 | 7.62 | 0.008 |
| VEGFA | 105.75 | 109.40 | 129.00 | 206.65 | 0.625 |
| VEGFD | 12.98 | 15.04 | 10.82 | 7.39 | 0.154 |

Variables are represented as median and IQR (interquartile range). N, number of patients

**Supplemental Table 1a**: Cytokine level analyses according to ABO blood group at first moment by using the Mann Whitney U test.

| **Second (b)** | **Group O**  **(N=24)** | | **Group A/B/AB**  **(N=62)** | | ***p*** |
| --- | --- | --- | --- | --- | --- |
|  | **Median** | **IQR** | **Median** | **IQR** |  |
| BDNF | 63.42 | 362.83 | 42.76 | 80.50 | 0.101 |
| EGF | 2.18 | 5.30 | 1.57 | 2.99 | 0.693 |
| Eotaxin | 14.52 | 12.22 | 13.70 | 11.12 | 0.482 |
| GMCSF | 16.85 | 35.98 | 10.43 | 23.67 | 0.141 |
| GROa | 3.03 | 2.64 | 2.05 | 1.54 | 0.583 |
| HGF | 240.25 | 302 | 337 | 637.50 | 0.053 |
| IFNa | 0.30 | 0.36 | 0.22 | 0.30 | 0.538 |
| IFNg | 8.80 | 5.36 | 8.19 | 6.98 | 0.904 |
| IL1a | 1.70 | 5.61 | 2.12 | 7.64 | 0.862 |
| IL1b | 8.23 | 7.82 | 5.52 | 6.40 | 0.044 |
| IL10 | 1.47 | 1.46 | 1.34 | 1.15 | 0.665 |
| IL13 | 2.10 | 1.90 | 1.94 | 2.44 | 0.668 |
| IL15 | 19.60 | 14.86 | 13.25 | 13.94 | 0.017 |
| IL17a | 10.38 | 14.54 | 4.86 | 9.10 | 0.016 |
| IL18 | 50.37 | 50.61 | 43.38 | 55.51 | 0.927 |
| IL1RA | 471.25 | 1440.88 | 469.98 | 920 | 0.402 |
| IL2 | 22.65 | 19.45 | 11.13 | 17.93 | 0.004 |
| IL22 | 4.23 | 9.91 | 2.15 | 13.92 | 0.679 |
| IL27 | 19.38 | 43.40 | 15.93 | 44.31 | 0.802 |
| IL4 | 5.66 | 6.05 | 4.98 | 4.09 | 0.506 |
| IL5 | 6.11 | 36.11 | 3.22 | 4.94 | 0.166 |
| IL6 | 10.06 | 32.24 | 8.91 | 20.95 | 0.583 |
| IL7 | 2.20 | 2.40 | 1.30 | 2.20 | 0.089 |
| IL8 | 2.20 | 10.83 | 2.13 | 8.93 | 0.693 |
| IP1b | 40.80 | 41.51 | 39.80 | 49.39 | 0.981 |
| IP10 | 15.75 | 12.76 | 18.50 | 16.68 | 0.233 |
| LIF | 17.30 | 25.92 | 10.16 | 11.24 | 0.136 |
| MCP1 | 32.60 | 38.01 | 31.22 | 36.41 | 0.795 |
| MIP1a | 4.49 | 10.03 | 2.28 | 7.48 | 0.151 |
| PDGFBB | 816.25 | 1694.63 | 338.75 | 718.50 | 0.055 |
| PIGF1 | 20.09 | 105.34 | 10.70 | 73.56 | 0.453 |
| RANTES | 34.60 | 15.75 | 26.73 | 20.86 | 0.102 |
| SCF | 5.82 | 9.99 | 6.98 | 11.04 | 0.946 |
| SDF1a | 539.25 | 861.75 | 591.75 | 631.12 | 0.857 |
| TNFa | 7.85 | 16.35 | 5.13 | 8.61 | 0.045 |
| VEGFA | 92.25 | 110.95 | 96.98 | 252.58 | 0.494 |
| VEGFD | 13.48 | 12.86 | 9.84 | 7.52 | 0.111 |

Variables are represented as median and IQR (interquartile range). N, number of patients

**Supplemental Table 1b**: Cytokine level analyses according to ABO blood group at second moment by using the Mann Whitney U test.
