# Supplementary material for: Can the Cytokine Profile According to ABO Blood Groups Be Related to Worse Outcome in COVID-19 Patients? Yes, They Can
Source: Front Immunol. 2021 Oct 13;12:726283. doi: 10.3389/fimmu.2021.726283 (PMC8548690; doi:10.3389/fimmu.2021.726283)
Supplement: Supplementary file 3 [file Table_2.docx]

|  | **Blood Group O** | | | **Group A/B/AB** | | |
| --- | --- | --- | --- | --- | --- | --- |
|  | **Median** | | **Difference**  **(%)** | **Median** | | **Difference**  **(%)** |
|  | **“A”**  **n=35** | **“B”**  **n=24** |  | **“A”**  **n=73** | **“B”**  **n=62** |  |
| **BDNF** | 103.80 | 63.42 | - 40 % | 45.75 | 42.76 | - 7 % |
| **EGF** | 2.88 | 2.18 | - 25 % | 1.45 | 1.57 | + 8 % |
| **GMCSF** | 21.98 | 16.85 | - 24 % | 11.46 | 10.43 | - 9 % |
| **HGF** | 149.00 | 240.25 | + 60 % | 316.50 | 337 | + 6 % |
| **IFNa** | 0.48 | 0.30 | - 38 % | 0.23 | 0.22 | - 5 % |
| **IL1b** | 10.01 | 8.23 | - 18 % | 5.99 | 5.52 | - 10 % |
| **IL13** | 3.51 | 2.10 | - 40 % | 1.89 | 1.94 | + 3 % |
| **IL15** | 17.30 | 19.60 | + 13 % | 11.65 | 13.25 | + 14 % |
| **IL17a** | 11.60 | 10.38 | - 11 % | 5.77 | 4.86 | - 16 % |
| **IL2** | 20.23 | 22.65 | + 12 % | 14.20 | 11.13 | - 22 % |
| **IL4** | 7.44 | 5.66 | - 24 % | 5.09 | 4.98 | - 2 % |
| **IL5** | 8.82 | 6.11 | - 31 % | 4.10 | 3.22 | - 20 % |
| **IL7** | 2.38 | 2.20 | - 8 % | 1.50 | 1.30 | - 13 % |
| **LIF** | 20.60 | 17.30 | - 16 % | 11.01 | 10.16 | - 8 % |
| **MIP1a** | 5.62 | 4.49 | - 20 % | 2.53 | 2.28 | - 10 % |
| **TNFa** | 11.98 | 7.85 | - 35 % | 6.52 | 5.13 | - 11 % |

**Supplemental Table 2**: Evolution of the level of significant cytokines expressed by medians and their percentage of difference. “A”: Determination of cytokines after admission to hospital Ward or Intensive Care Unit. “B”: Determination of cytokines sixth day after hospital admission. ICU; Intensive Care Unit.
