# Supplementary material for: Can the Cytokine Profile According to ABO Blood Groups Be Related to Worse Outcome in COVID-19 Patients? Yes, They Can
Source: Front Immunol. 2021 Oct 13;12:726283. doi: 10.3389/fimmu.2021.726283 (PMC8548690; doi:10.3389/fimmu.2021.726283)
Supplement: Supplementary file 4 [file Table_3.docx]

**Blood Group O**

**First moment (a)**

(Admission to hospital Ward or Intensive Care Unit)

| **First**  **N=35** | **Non outcome**  **(N=27)** | | **Bad outcome**  **(N=8)** | | ***p*** |
| --- | --- | --- | --- | --- | --- |
|  | **Median** | **IQR** | **Median** | **IQR** |  |
| BDNF | 96.55 | 326.97 | 116.05 | 237.95 | 0.968 |
| EGF | 3.43 | 11.05 | 4.41 | 16.66 | 0.871 |
| Eotaxin | 16.39 | 8.31 | 10.70 | 12.97 | 0.239 |
| GMCSF | 16.14 | 64.07 | 27.6 | 31.56 | 0.516 |
| GROa | 2.84 | 2.9 | 3.23 | 5.03 | 0.167 |
| HGF | 126.52 | 183.87 | 345.25 | 396.75 | 0.015 |
| IFNa | 0.41 | 0.71 | 0.52 | 0.71 | 0.273 |
| IFNg | 9.87 | 7.48 | 10.34 | 12.06 | 0.330 |
| IL1a | 2.20 | 5.25 | 4.50 | 5.93 | 0.291 |
| IL1b | 10.00 | 13.21 | 10.16 | 9.44 | 0.516 |
| IL10 | 1.58 | 1.85 | 2.33 | 2.66 | 0.144 |
| IL13 | 3.51 | 7.75 | 3.09 | 11.76 | 0.951 |
| IL15 | 17.30 | 23.28 | 17.50 | 14.16 | 0.776 |
| IL17a | 9.36 | 17.13 | 15.73 | 6.40 | 0.394 |
| IL18 | 43.45 | 51.25 | 68.50 | 38.16 | 0.133 |
| IL1RA | 735.75 | 162.87 | 1385.00 | 2527.38 | 0.123 |
| IL2 | 18.90 | 23.28 | 20.28 | 26.61 | 0.490 |
| IL22 | 6.45 | 15.6 | 7.09 | 35.85 | 0.465 |
| IL27 | 30.66 | 106.60 | 22.49 | 164.87 | 0.968 |
| IL4 | 6.77 | 9.77 | 7.78 | 5.65 | 0.490 |
| IL5 | 7.30 | 30.42 | 38.03 | 47.04 | 0.109 |
| IL6 | 8.12 | 28.01 | 35.78 | 39.63 | 0.113 |
| IL7 | 2.22 | 3.28 | 2.61 | 3.33 | 0.570 |
| IL8 | 2.51 | 6.49 | 2.06 | 9.25 | 0.808 |
| IP1b | 49.25 | 32.74 | 50.75 | 59.64 | 0.903 |
| IP10 | 20.00 | 22.29 | 20.38 | 51.73 | 0.543 |
| LIF | 12.97 | 21.84 | 26.18 | 19.73 | 0.071 |
| MCP1 | 32.28 | 33.10 | 36.25 | 55.48 | 0.330 |
| MIP1a | 3.91 | 12.25 | 7.66 | 10.98 | 0.685 |
| PDGFBB | 339.00 | 795.13 | 880.00 | 1841.63 | 0.028 |
| PIGF1 | 4.41 | 29.30 | 42.13 | 138.52 | 0.161 |
| RANTES | 27.23 | 22.81 | 30.53 | 35.45 | 0.968 |
| SCF | 7.32 | 6.27 | 9.95 | 19.78 | 0.477 |
| SDF1a | 562.33 | 552.88 | 768.00 | 2627.63 | 0.761 |
| TNFa | 8.65 | 13.23 | 16.50 | 12.86 | 0.155 |
| VEGFA | 99.30 | 114.04 | 139.00 | 285.15 | 0.273 |
| VEGFD | 12.98 | 16.77 | 12.51 | 16.11 | 0.903 |

Bad outcome means intubation or death. Variables are represented as median and IQR (interquartile range). N, number of patients.

**Supplemental Table 3a**: Cytokine level analyses according to outcome in O blood group at first moment by using the Mann Whitney U test.

**Blood Group O**

**Second moment (b)**

(Sixth day after hospital admission)

| **Second**  **N=24** | **Non outcome**  **(N=16)** | | **Bad outcome**  **(N=8)** | | ***p*** |
| --- | --- | --- | --- | --- | --- |
|  | **Median** | **IQR** | **Median** | **IQR** |  |
| BDNF | 59.75 | 406.83 | 87.70 | 206.91 | 0.951 |
| EGF | 1.25 | 5.17 | 2.66 | 11.54 | 0.098 |
| Eotaxin | 15.25 | 10.60 | 10.45 | 14.28 | 0.426 |
| GMCSF | 9.83 | 34.87 | 38.40 | 47.55 | 0.126 |
| GROa | 2.18 | 1.78 | 3.01 | 1.52 | 0.050 |
| HGF | 131.50 | 307.44 | 285.25 | 876.25 | 0.245 |
| IFNa | 0.19 | 0.31 | 0.48 | 0.95 | 0.023 |
| IFNg | 8.20 | 5.17 | 11.17 | 12.00 | 0.043 |
| IL1a | 0.85 | 5.13 | 3.70 | 11.46 | 0.086 |
| IL1b | 7.23 | 5.70 | 12.73 | 12.12 | 0.221 |
| IL10 | 1.29 | 0.77 | 2.45 | 1.78 | 0.012 |
| IL13 | 1.79 | 1.66 | 2.58 | 8.96 | 0.050 |
| IL15 | 18.15 | 15.33 | 21.50 | 18.14 | 0.903 |
| IL17a | 7.56 | 10.62 | 15.78 | 17.06 | 0.245 |
| IL18 | 44.45 | 60.98 | 50.63 | 46.58 | 0.806 |
| IL1RA | 397.25 | 898.92 | 2138.28 | 1546.00 | 0.098 |
| IL2 | 20.45 | 18.00 | 27.55 | 23.67 | 0.408 |
| IL22 | 2.26 | 7.62 | 7.58 | 120.14 | 0.086 |
| IL27 | 26.08 | 30.93 | 40.06 | 124.42 | 0.023 |
| IL4 | 4.58 | 4.74 | 8.82 | 13.05 | 0.066 |
| IL5 | 3.56 | 16.68 | 48.28 | 114.03 | 0.027 |
| IL6 | 7.59 | 10.02 | 23.96 | 34.40 | 0.126 |
| IL7 | 1.81 | 2.30 | 2.78 | 2.29 | 0.270 |
| IL8 | 1.85 | 12.27 | 2.49 | 8.48 | 0.624 |
| IP1b | 43.58 | 41.29 | 33.15 | 43.52 | 0.245 |
| IP10 | 15.75 | 12.71 | 16.00 | 14.21 | 0.903 |
| LIF | 9.60 | 12.57 | 32.20 | 18.30 | 0.012 |
| MCP1 | 29.95 | 41.08 | 32.60 | 42.01 | 0.759 |
| MIP1a | 2.94 | 10.56 | 6.83 | 8.56 | 0.198 |
| PDGFBB | 544.25 | 897.88 | 1560.00 | 2719.50 | 0.076 |
| PIGF1 | 2.62 | 73.57 | 81.75 | 122.93 | 0.050 |
| RANTES | 30.60 | 8.49 | 34.20 | 122.16 | 0.951 |
| SCF | 5.60 | 10.00 | 10.36 | 10.34 | 0.951 |
| SDF1a | 539.25 | 340.38 | 547.50 | 3364.75 | 0.668 |
| TNFa | 7.12 | 8.41 | 18.25 | 20.56 | 0.178 |
| VEGFA | 84.85 | 103.45 | 117.10 | 253.85 | 0.668 |
| VEGFD | 13.48 | 12.88 | 15.85 | 15.45 | 0.903 |

Bad outcome means intubation or death. Variables are represented as median and IQR (interquartile range). N, number of patients.

**Supplemental Table 3b**: Cytokine level analyses according to outcome in O blood group at second moment by using the Mann Whitney U test.

**Blood Group A/B/AB**

**First moment (c)**

(Admission to hospital Ward or Intensive Care Unit)

| **First**  **N=73** | **Non outcome**  **(N=41)** | | **Bad outcome**  **(N=32)** | | ***p*** |
| --- | --- | --- | --- | --- | --- |
|  | **Median** | **IQR** | **Median** | **IQR** |  |
| BDNF | 45.75 | 129.73 | 43.95 | 114.09 | 0.601 |
| EGF | 1.21 | 2.46 | 1.62 | 3.41 | 0.594 |
| Eotaxin | 12.55 | 9.58 | 12.17 | 10.86 | 0.938 |
| GMCSF | 12.83 | 23.5 | 11.27 | 8.16 | 0.722 |
| GROa | 2.12 | 2.08 | 2.45 | 3.5 | 0.436 |
| HGF | 185.50 | 196.95 | 698.88 | 879.75 | <0.001 |
| IFNa | 0.23 | 0.45 | 0.24 | 0.39 | 0.714 |
| IFNg | 8.09 | 5.44 | 9.32 | 12.46 | 0.114 |
| IL1a | 2.54 | 11.08 | 2.37 | 4.18 | 0.362 |
| IL1b | 7.00 | 7.43 | 5.24 | 5.32 | 0.070 |
| IL10 | 1.33 | 0.73 | 1.35 | 2.28 | 0.491 |
| IL13 | 1.85 | 2.65 | 1.89 | 1.65 | 0.991 |
| IL15 | 13.40 | 10.16 | 9.61 | 17.02 | 0.404 |
| IL17a | 5.90 | 8.72 | 5.60 | 8.72 | 0.505 |
| IL18 | 34.60 | 59.68 | 63.00 | 90.42 | 0.048 |
| IL1RA | 454.50 | 526.00 | 640.50 | 2180.19 | 0.129 |
| IL2 | 14.55 | 17.14 | 12.28 | 13.72 | 0.145 |
| IL22 | 1.82 | 15.72 | 2.47 | 13.20 | 0.894 |
| IL27 | 17.59 | 69.78 | 14.08 | 21.20 | 0.312 |
| IL4 | 4.91 | 4.56 | 5.32 | 3.68 | 0.911 |
| IL5 | 3.01 | 6.82 | 5.08 | 14.13 | 0.312 |
| IL6 | 9.16 | 12.46 | 7.49 | 16.27 | 0.705 |
| IL7 | 1.53 | 1.68 | 1.50 | 2.16 | 0.356 |
| IL8 | 2.00 | 5.22 | 1.64 | 3.82 | 0.411 |
| IP1b | 47.70 | 39.20 | 59.70 | 64.24 | 0.697 |
| IP10 | 22.25 | 28.87 | 32.20 | 52.83 | 0.058 |
| LIF | 13.35 | 10.80 | 8.97 | 15.99 | 0.359 |
| MCP1 | 27.00 | 20.90 | 47.63 | 38.18 | <0.001 |
| MIP1a | 2.95 | 7.35 | 2.00 | 8.97 | 0.956 |
| PDGFBB | 364.00 | 1176.90 | 509.00 | 1065.36 | 0.296 |
| PIGF1 | 4.76 | 69.06 | 10.28 | 70.46 | 0.859 |
| RANTES | 27.20 | 29.03 | 24.40 | 23.50 | 0.824 |
| SCF | 6.60 | 4.97 | 9.01 | 13.66 | 0.252 |
| SDF1a | 625.50 | 633.00 | 737.50 | 966.00 | 0.541 |
| TNFa | 6.61 | 7.60 | 6.08 | 11.45 | 0.582 |
| VEGFA | 119.55 | 223.17 | 134.62 | 229.26 | 0.807 |
| VEGFD | 11.60 | 7.83 | 9.96 | 8.14 | 0.100 |

Bad outcome means intubation or death. Variables are represented as median and IQR (interquartile range). N, number of patients.

**Supplemental Table 3c**: Cytokine level analyses according to outcome in A/B/AB blood group at first moment by using the Mann Whitney U test.

**Blood Group A/B/AB**

**Second moment (d)**

(Sixth day after hospital admission)

| **Second**  **N=62** | **Non outcome**  **(N=33)** | | **Bad outcome**  **(N=29)** | | ***p*** |
| --- | --- | --- | --- | --- | --- |
|  | **Median** | **IQR** | **Median** | **IQR** |  |
| BDNF | 46.50 | 69.26 | 36.70 | 105.05 | 0.563 |
| EGF | 1.41 | 5.57 | 1.61 | 1.74 | 0.816 |
| Eotaxin | 13.10 | 10.39 | 16.25 | 11.27 | 0.230 |
| GMCSF | 14.35 | 33.54 | 9.63 | 12.42 | 0.077 |
| GROa | 1.81 | 1.41 | 2.21 | 1.89 | 0.355 |
| HGF | 187.00 | 221.17 | 855.00 | 998.25 | <0.001 |
| IFNa | 0.17 | 0.22 | 0.27 | 0.39 | 0.156 |
| IFNg | 7.78 | 5.75 | 9.39 | 8.96 | 0.233 |
| IL1a | 2.38 | 7.76 | 1.44 | 7.93 | 0.667 |
| IL1b | 7.27 | 7.40 | 4.51 | 5.44 | 0.035 |
| IL10 | 1.21 | 1.28 | 1.43 | 1.12 | 0.165 |
| IL13 | 2.10 | 4.61 | 1.93 | 1.66 | 0.160 |
| IL15 | 14.60 | 13.96 | 10.19 | 14.43 | 0.116 |
| IL17a | 5.91 | 10.39 | 4.21 | 7.47 | 0.133 |
| IL18 | 32.70 | 32.49 | 70.35 | 66.88 | 0.036 |
| IL1RA | 473.00 | 732.30 | 466.97 | 1406.60 | 0.682 |
| IL2 | 14.75 | 23.00 | 8.32 | 9.18 | 0.067 |
| IL22 | 1.67 | 24.11 | 3.94 | 11.44 | 0.467 |
| IL27 | 18.12 | 84.04 | 15.84 | 21.31 | 0.268 |
| IL4 | 4.25 | 3.95 | 5.16 | 4.20 | 0.636 |
| IL5 | 2.67 | 4.25 | 4.15 | 5.84 | 0.187 |
| IL6 | 9.00 | 17.23 | 8.82 | 30.89 | 0.667 |
| IL7 | 1.37 | 2.31 | 1.14 | 1.95 | 0.320 |
| IL8 | 2.17 | 14.76 | 1.96 | 6.47 | 0.709 |
| IP1b | 39.85 | 46.08 | 39.75 | 55.05 | 0.949 |
| IP10 | 17.60 | 14.13 | 18.95 | 21.48 | 0.489 |
| LIF | 11.60 | 14.63 | 9.52 | 6.90 | 0.256 |
| MCP1 | 23.00 | 16.23 | 49.35 | 45.60 | 0.002 |
| MIP1a | 2.98 | 7.82 | 1.97 | 5.56 | 0.459 |
| PDGFBB | 325.00 | 588.75 | 355.00 | 745.50 | 0.772 |
| PIGF1 | 6.17 | 43.53 | 14.14 | 87.23 | 0.549 |
| RANTES | 29.30 | 26.28 | 26.25 | 14.78 | 0.386 |
| SCF | 6.89 | 6.27 | 7.44 | 18.47 | 0.225 |
| SDF1a | 485.50 | 462.00 | 917.50 | 1076.50 | 0.016 |
| TNFa | 5.13 | 9.17 | 5.20 | 7.45 | 0.938 |
| VEGFA | 82.15 | 212.10 | 165.50 | 254.92 | 0.320 |
| VEGFD | 12.50 | 9.42 | 9.25 | 6.79 | 0.022 |

Bad outcome means intubation or death. Variables are represented as median and IQR (interquartile range). N, number of patients.

**Supplemental Table 3d**: Cytokine level analyses according to outcome in A/B/AB blood group at second moment by using the Mann Whitney U test.
